# Using approximate Bayesian computation to quantify cell–cell adhesion parameters in a cell migratory process

**DOI:** 10.1038/s41540-017-0010-7

**Published:** 2017-03-10

**Authors:** Robert J. H. Ross, R. E. Baker, Andrew Parker, M. J. Ford, R. L. Mort, C. A. Yates

**Affiliations:** 1 Wolfson Centre for Mathematical Biology, Mathematical Institute, University of Oxford, Radcliffe Observatory Quarter, Woodstock Road, Oxford, OX2 6GG UK; 2 MRC Human Genetics Unit, MRC IGMM, Western General Hospital, University of Edinburgh, Edinburgh, EH4 2XU UK; 3 Division of Biomedical and Life Sciences, Faculty of Health and Medicine, Furness Building, Lancaster University, Bailrigg, Lancaster LA1 4YG UK; 4 Department of Mathematical Sciences, Centre for Mathematical Biology, University of Bath, Claverton Down, Bath BA2 7AY UK

## Abstract

In this work, we implement approximate Bayesian computational methods to improve the design of a wound-healing assay used to quantify cell–cell interactions. This is important as cell–cell interactions, such as adhesion and repulsion, have been shown to play a role in cell migration. Initially, we demonstrate with a model of an *unrealistic* experiment that we are able to identify model parameters that describe agent motility and adhesion, given we choose appropriate summary statistics for our model data. Following this, we replace our model of an unrealistic experiment with a model representative of a practically realisable experiment. We demonstrate that, given the current (and commonly used) experimental set-up, our model parameters cannot be accurately identified using approximate Bayesian computation methods. We compare new experimental designs through simulation, and show more accurate identification of model parameters is possible by expanding the size of the domain upon which the experiment is performed, as opposed to increasing the number of experimental replicates. The results presented in this work, therefore, describe time *and* cost-saving alterations for a commonly performed experiment for identifying cell motility parameters. Moreover, this work will be of interest to those concerned with performing experiments that allow for the accurate identification of parameters governing cell migratory processes, especially cell migratory processes in which cell–cell adhesion or repulsion are known to play a significant role.

## Math heals: Using computational methods to improve design of wound healing assay

Cell motility is a central process in wound healing and relies on complex cell-cell interactions. A team of mathematicians led by Ruth Baker and Kit Yates at the University of Oxford utilised computer simulations to re-design wound-healing assays that efficiently identify cell motility parameters. New experimental designs through computer simulation can more accurately identify cell motility parameters by expanding the size of the domain upon which the experiment is performed, as opposed to increasing the number of experimental replicates. The results describe time and cost-saving alterations for an experimental method for evaluate complex cell-cell interactions.

## Introduction

Cell–cell interactions are known to play an important role in several cell migration processes. For example, multiple different cell–cell interactions, such as cell–cell signalling and cell–cell adhesion,^1^ have been identified as promoting metastasis in breast cancer. Repulsive interactions mediated via ephrins on the surface of neural crest stem cells are known to coordinate the early stages of melanoblast migration away from the neural tube.^2^ More fundamentally, it is hypothesised that the emergence of cell–cell interactions over one billion years ago helped establish the necessary conditions for multicellular organisms.^3^

A well-established approach for studying cell migration is to construct an agent-based model (ABM) to simulate the cell migratory process of interest.^4–8^ Typically, this involves using a computational model to simulate a population of agents on a two-dimensional surface, or in a three-dimensional volume. The agents in the ABM represent cells, and each agent is able to move and interact with other agents in the ABM. In this work, we use an ABM to simulate a wound-healing assay (Wound-healing assays are also often referred to as scratch assays.), an experiment commonly used for studying cell motility.^9–15^ Other modelling approaches apart from ABMs have been employed to study wound-healing. For instance, a huge amount of research has been completed using continuum methods to model the wound-healing process (see Flegg *et al*.^16^ for a recent review of the field). However, we employ an ABM in this work because they provide an intuitive representation of cells, and allow for complex behaviours representing biological processes, such as cell–cell interactions and volume exclusion, to be easily assigned to agents in the ABM.

If an ABM is an *effective* (By an effective representation, we mean the ABM captures the salient features of the process of interest, and is, therefore, a viable research tool with which to study the process of interest.) representation of a cell migration process it can be used for a number of purposes. One such purpose for an ABM is to perform in silico experiments to test scientific hypotheses. For instance, a recent study used an ABM to demonstrate that a simple mechanism of undirected cell movement and proliferation could account for neural crest stem cell colonisation of the developing epidermis in the embryonic mouse.^4^ Other studies involving ABMs have tested hypotheses concerning the influence of matrix stiffness and matrix architecture on cell migration,^17^ and the mechanism by which cranial neural crest stem cells become ‘leaders’ or ‘followers’ in the embryonic chick to facilitate their collective migration.^6–8^

ABMs can also be used to *identify* parameters in experimental data (with the caveat that the parameters are model-dependent). The reasoning behind using an ABM to identify parameters in experimental data is as follows: if an ABM is an effective representation of an experiment, then the parameter values the ABM requires to reproduce the experimental data may be representative of the parameter values in the biological process that is the focus of the experiment. For instance, the value of a parameter that describes cell proliferation rate. Even if the parameter values in the parameterised ABM are not representative of the parameter values in the biological process, the parameterised ABM may still be used to make predictions about the process of interest by performing in silico experiments, as described above. These predictions can then be experimentally tested.

Alternatively, if the ABM is an effective representation of an experiment (i.e., the experimental data can be reproduced), but the parameters of the ABM are not identifiable, this may suggest the experiment is not well-designed (that is, if the experiment has been designed to estimate parameters). By parameters not being identifiable we mean that different parameter values in the ABM can reproduce the same experimental data. If this is the case, the ABM can then be used to suggest improvements to the experiment’s design, namely by altering the ABM design such that the ABM parameters become identifiable. These alterations can then be applied to the experiment to improve parameter identifiability. For example, a recent study using an ABM has examined the time-points at which data should be collected from an experiment to maximise the identifiability of ABM parameters.^11^ Other theoretical work has shown how to maximise the information content of an experiment by choosing an appropriate experimental set-up.^18^

The focus of our study is to determine the experimental conditions, and experimental data, required for the accurate identification of cell motility and adhesion parameters in an ABM of a wound-healing assay. To do so, we employ approximate Bayesian computation (ABC), a probabilistic approach whereby a probability distribution for the parameter(s) of interest is estimated, as opposed to a point estimate.^10, 19, 20^ Although ABC is well-established in some fields, for instance in population genetics,^21^ its applicability for ABMs representing cell migration is still an area of active research.^9–11, 22–24^ Recent studies combining ABC and ABMs have been able to identify motility and proliferation rates in cell migratory processes,^10^ and improve the experimental design of scratch assays.^11^ However, as far as we are aware no study to date has used ABC methods to examine the experimental conditions, and experimental data, required for the accurate identification of cell motility and adhesion parameters in a wound-healing assay.

Other methods to identify parameters from experimental data using ABMs also exist. For instance, a standard approach is to generate point estimates of model parameters that best reproduce statistics of the experimental data in the ABM. For example, the generation of motility and proliferation rates for agents in an ABM representing a biological process.^4^ This approach, while applicable in some circumstances, often gives little insight into how much uncertainty exists in the parameters chosen, a factor that can be of importance when analysing biological systems. For example, relationships between parameter uncertainty and system robustness are thought to be connected in biological function at a systems level.^25^

The outline of this work is as follows: in the Methods Section we introduce our ABM and define the cell–cell interactions we implement. We also outline the method of ABC, and the summary statistics we use to analyse the ABM output. In the Results Section we present results and demonstrate that, given an ABM representing an unrealistic experiment, we are able to identify ABM parameters for agent motility and adhesion. Following this, we replace our ABM representing an unrealistic experiment with an ABM that simulates a practically realisable experiment. In doing so, we show that agent motility and adhesion parameters cannot be successfully identified using ABC given the current experimental design. To improve parameter identifiability we compare different experimental set-ups, and show that identification of ABM parameters is made more accurate if the size of the domain upon which the experiment is performed is expanded, as opposed to the number of experimental replicates increased. Experimentally, expanding the size of the domain is equivalent to increasing the field of view of the microscope used to collect the experimental data. For instance, generating five experimental replicates on a larger domain enables more accurate identification of ABM parameters than generating 500 experimental replicates on a domain eight times smaller. Finally, we discuss the results presented in this work.

## Results

We begin by demonstrating that for an ABM representing an unrealistic experiment we are able to identify model parameters, given appropriate summary statistics.

### Unrealistic experiment

To ascertain the effectiveness of the chosen summary statistics to identify model parameters, we attempt to identify *Θ* from data generated *synthetically*. Synthetic data is ABM data generated with fixed parameter values, and so can be thought of as a simulation equivalent of experimental data. To generate the synthetic data using the ABM we proceed as follows:

1. We choose parameters *Θ* to identify. To help clarify this explanation let us make these parameters *Θ* = (*P*_*m*_, *α*) = (0.5,0.1) in model A (A value of *P*_*m*_ = 0.5, given that the simulation time will later be defined to be in minutes, and the length of a lattice site represents cell length (typically between 10–100 μm, means that the motility of the agents is biologically realistic. The parameter *α* is dimensionless. The experimental realism of these parameters will be expanded on when we address the simulation of a practically realisable experiment.).
2. For model A we perform a simulation of the ABM with *Θ* = (0.5,0.1), generate data, *D*, and calculate summary statistics, *S*(*D*), from the simulation at our time-points of interest. These times are *t* = [240,480,720]. We choose these times as they are the times (in minutes) we will later analyse for the simulations of the practically realisable experiment, and correspond to 4, 8 and 12 h into an experiment.
3. We repeat step 2 ten times and calculate the ensemble average for each summary statistic for each individual time-point.

This procedure generates synthetic data for which we will now attempt to identify the parameters. In this work, we present representative results using *P*_*m*_ = 0.5 and *α* = 0.1 for model A, and *P*_*m*_ = 0.5 and *α* = 0.25, and *P*_*m*_ = 0.5 and *α* = −0.1 for model B.

Throughout this work, we sample *P*_*m*_ and *α* for our model from uniform priors. In the case of model A, *P*_*m*_ ∈ [0, 1] and *α* ∈ [−0.2, 0.25], and for model B, *P*_*m*_ ∈ [0, 1] and *α* ∈ [−0.2, 1.0]. We stipulate these lower and upper bounds for *α* for both models A and B to make sure inequalities (2) and (4) are satisfied.

We begin by implementing an ABC rejection algorithm that proceeds as follows:

1. Run 10^4^ ABM simulations, in each case using *Θ* sampled uniformly at random from the prior distribution.

2. Compute the distance *d* as defined in Eq. 13 for simulation times *t* = [240,480,720].

3. Accept the 100 parameter values, *Θ*, that give the smallest values of *d*.

**Fig. 1:**
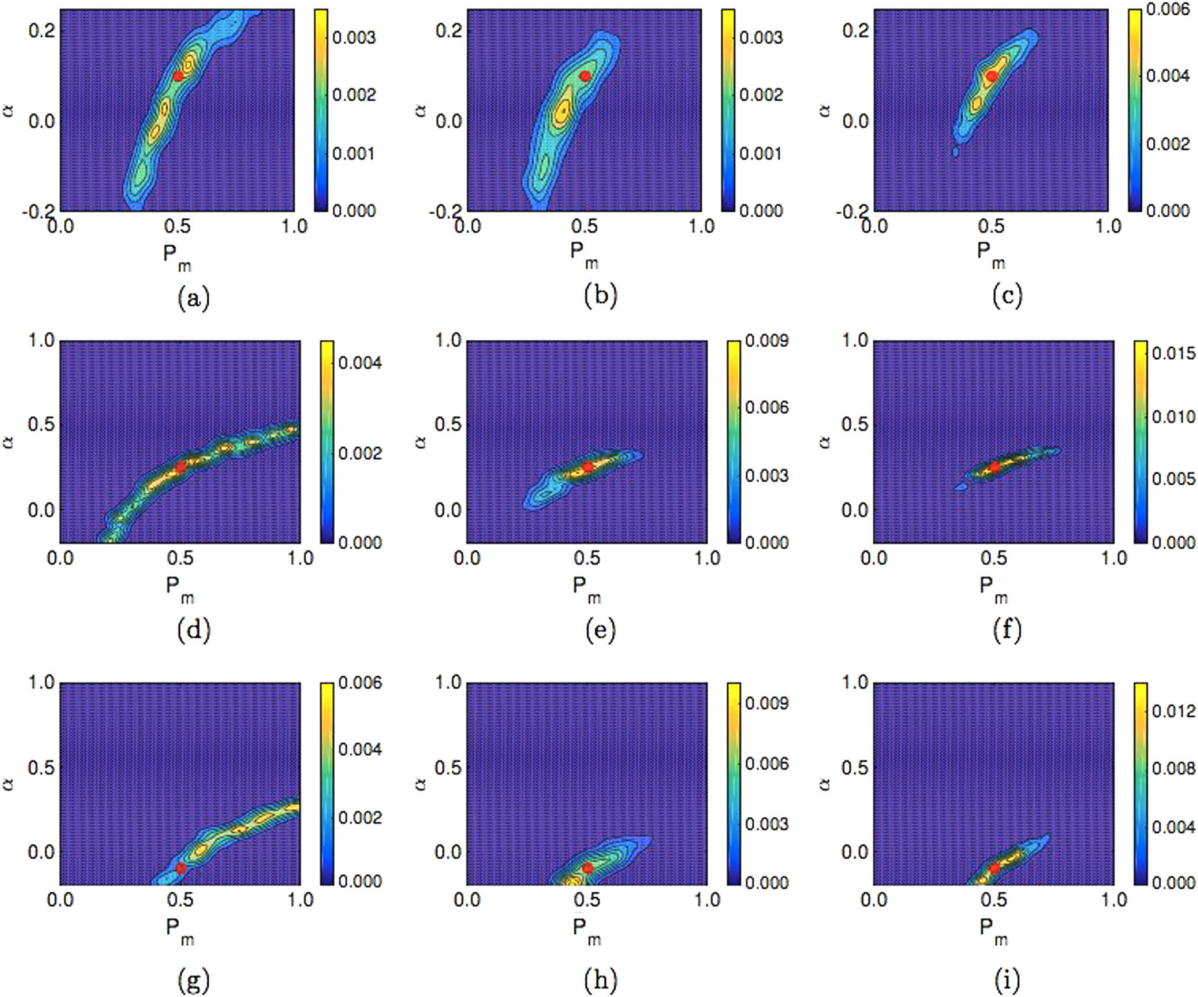
**a–c** Posterior distributions for model A for an unrealistic experiment with different summary statistics: **a** average displacement of agents in the *horizontal* direction; **b** agent density profile; **c** PCF. In all cases the *red dot* indicates the value of the parameters used to generate the synthetic data, *P*_*m*_ = 0.5, *α* = 0.1. As indicated by the colour bar the *yellow regions* indicate areas of high relative density of the posterior distribution, while the *blue regions* indicate areas of low relative density of the posterior distribution. **d–f** Model B, *P*_*m*_ = 0.5, *α* = 0.25: **d** average displacement of agents in the *horizontal* direction; **e** agent density profile; **f** PCF. **g–i** Model B, *P*_*m*_ = 0.5, *α* = −0.1; **g** average displacement of agents in the horizontal direction; **h** agent density profile; **i** PCF

In Fig. 1, the posteriors generated using each of the three summary statistics applied to data from simulations of an unrealistic experiment are displayed. The most effective summary statistic for identifying the synthetic data parameters is the PCF. This is evident in the location of the posterior distribution density relative to the red dot (the red dot represents the synthetic data parameter values), and the narrow spread of the posterior distribution density as indicated by the scale bar in Fig. 1c, f and i. The agent density profile summary statistic performs less well than the PCF for parameter identification, especially for model A (Fig. 1b). In the case of the average agent displacement summary statistic many combinations of *P*_*m*_ and *α* lead to the same average agent displacement, which results in an extended region of possible parameter values. To some extent this is to be expected, as increasing either *P*_*m*_ or *α* will have opposing effects on the average agent displacement. This means that using agent displacement as a summary statistic results in parameter identifiability issues in this example.

To quantify the difference between the performance of the different summary statistics we use the Kullback-Leibler divergence (KLD), which is a measure of the information gained in moving from the prior distribution to the posterior distribution.^26^ The KLD for a discrete probability distribution is defined as follows:

1 $${D_{KL}}\left( {p{\rm{|}}\pi } \right) = {\sum_{l}}p \left( {{{\it{\Theta }}_l}{\rm{|}}D} \right)\log \left( {\frac{{p\left( {{{\it{\Theta }}_l}{\rm{|}}D} \right)}}{{\pi \left( {{{\it{\Theta }}_l}} \right)}}} \right),$$

where the index *l* accounts for all possible discretised parameter pairs (i.e., all combinations of *P*_*m*_ and *α*). A larger *D*_*KL*_(*p*|*π*) value suggests that more information is obtained (the entropy of the distribution is reduced) when moving from the prior distribution to the posterior distribution. However, this does not necessarily mean the posterior distribution is a more accurate representation of the parameter distribution. Therefore, the KLD should not be seen as ubiquitously applicable to inference problems similar to those described in this work. In particular, the KLD should be used with caution in scenarios in which an informative prior is used. In such scenarios, other methods to measure the improvement of an inference procedure have been examined and may be more suitable.^27^

To compute the KLD, we discretise our posterior distribution onto a lattice with 2^6^ equally spaced values of *P*_*m*_ and 2^6^ equally spaced values of *α*. Computing *D*_*KL*_(*p*|*π*) for all nine plots in Fig. 1 gives: (a) 1.77; (b) 1.70; (c) 2.32; and (d) 2.15; (e) 2.57; (f) 3.35; and (g) 2.45; (h) 2.72; (i) 3.27. In tandem with the proximity of the peak of the posterior distribution densities to the red dots in Fig. 1c, f and i, compared to Fig. 1a, b, d, e, g and h, this is increase in the KLD suggests that the PCF summary statistic is more effective for parameter identification than the average agent displacement and agent density profile summary statistics.

### Practically realisable experiment

In the previous section, we demonstrated that for unrealistic experimental conditions the PCF summary statistic is best able to identify synthetic data parameters (for data generated from an ABM of an unrealistic experiment), and so moving forward we will only use the PCF summary statistic for parameter identification. Previous work has combined summary statistics to improve parameter identification, and how best to combine summary statistics has been the focus of a significant amount of research, with a wide range of different methods examined.^10, 27–30^ However, in this case combining our summary statistics results in a negligible improvement to the posterior distribution (An example of a posterior distribution generated by combining all three summary statistics can be found in the supplementary material (Section S1)).

**Fig. 2:**
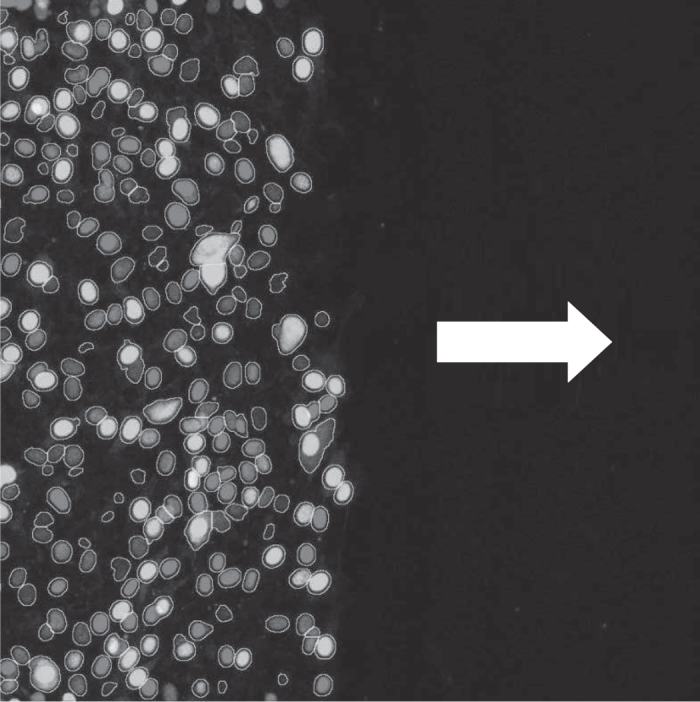
Typical initial frame of the experimental data. The cells are positioned such that they will migrate primarily horizontally into the space without cells, this space represents a wound (the direction of migration is indicated by the *white arrow*)

We now replace our ABM that represents an unrealistic experiment with an ABM that represents an actual experiment, and examine if synthetic data parameters can be identified in the ABM. That is, from this point on, we generate all synthetic data from an ABM based on a realistic experimental set-up. We provide brief details of the experiment here, however, a more detailed description can be found in the supplementary material (Section S2). In Fig. 2a typical initial frame of the experimental data can be seen.

In total, we have data from five replicates of the experiment. Therefore, we now generate our synthetic data from five replicates of the ABM, using the same procedure as described before. One key difference between the unrealistic and practically realisable experiments is the size of the domain and, because of this, the number of agents in a simulation.

The experimental images were captured by a microscope with a field of view of 597.24 µm by 597.24 µm. The cell size in the experimental images is consistent with each cell occupying a 26 µm by 26 µm square lattice site. Given the size of the microscope field of view this means the ABM domain size is *L*_*x*_ = 23 by *L*_*y*_ = 23. We use the average initial conditions from the experiment to generate the initial conditions in the ABM of a realistic experiment. Exact details of how the initial condition is generated in the ABM, and how experimental data is mapped to a lattice, can be found in the supplementary material (Section S3).

We also alter the ABM to have flux (nonperiodic) boundary conditions at the left-hand and right-hand boundaries of the domain (i.e., for lattice sites with *j* = 1 or *j* = *L*_*y*_). The left-most column is kept at or above a constant density throughout the simulation time course. That is, after any movement event from the left-most column in the simulation the column density of the left-most column is calculated, and if found to be below a certain density agents are added to empty sites in this column chosen uniformly at random until the required density is achieved. This mechanism ensures that the agent density profile in the ABM replicates the evolution of the experimental data throughout the simulation. Further details regarding the implementation of this boundary condition are provided in the supplementary material (Section S3). The top and bottom boundaries of the ABM domain remain periodic as cells were seen to move in and out of the microscope field at these boundaries in the experimental images, at an approximately equal rate.

To reduce computational time we now implement a Markov Chain Monte Carlo variant of ABC.^19^ Details of the implementation of the algorithm are given in the supplementary material (Section S4). As before we sample from uniform priors *P*_*m*_ ∈ [0, 1] and *α* ∈ [−0.2, 0.25] for model A, and *P*_*m*_ ∈ [0, 1] and *α* ∈ [−0.2, 1.0] for model B, and collect simulation data at *t* = [240,480,720]. We collect simulation data at three time-points so that the computational time is of practical length (our longest ABC Markov Chain Monte Carlo implementations took approximately 192 h). A value of *P*_*m*_ = 0.5, given that the simulation time is in minutes, and the length of a lattice site is 26 µm, means that the motility of the agents is biologically realistic. To be precise, the agents here are approximately five times faster than cell motility rates previously published (Using the relationship that the diffusion coefficient is equal to *P*_*m*_Δ^2^.) (refs 4, 9). However, the cells considered in refs 4, 9 are not thought to exhibit cell–cell adhesion, and so a higher motility rate for the agents is sensible as agent movement is reduced by cell–cell adhesion in our ABM.

**Fig. 3:**
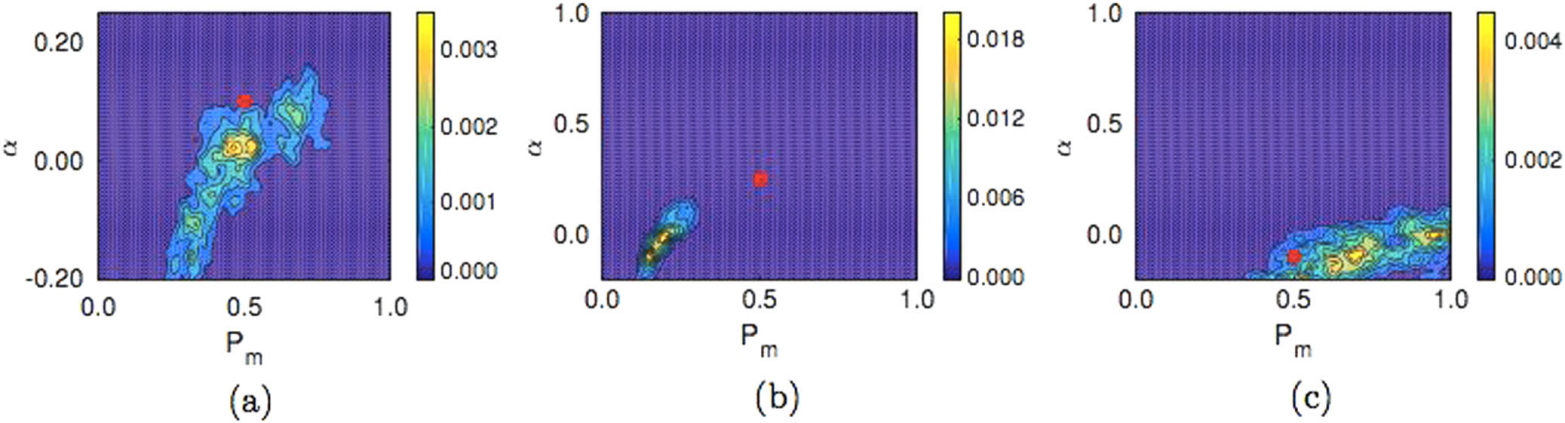
Posterior distributions for simulations of the realistic experiment described using the PCF as a summary statistic for an ABM of dimension *L*_*x*_ = 23 and *L*_*y*_ = 23. The synthetic data is generated from five replicates of the ABM. **a** Model A: *P*_*m*_ = 0.5, *α* = 0.1, **b** model B: *P*_*m*_ = 0.5, *α* = 0.25, **c** model B: *P*_*m*_ = 0.5, *α* = −0.1. In all cases the *red dot* indicates the value of the parameters used to generate synthetic data

In Fig. 3 it can be seen that the synthetic data parameters cannot be accurately identified using ABC, with the PCF summary statistic, given the current ABM design. This is evident in the location of the red dots (indicating the parameter values used to generate the synthetic data) relative to the posterior distributions, and the wide spread of the posterior distributions (indicated by the scale bar in Fig. 3). We have included the ABC Markov chain Monte Carlo traces corresponding to Fig. 3 in the supplementary material (Section S5).

**Fig. 4:**
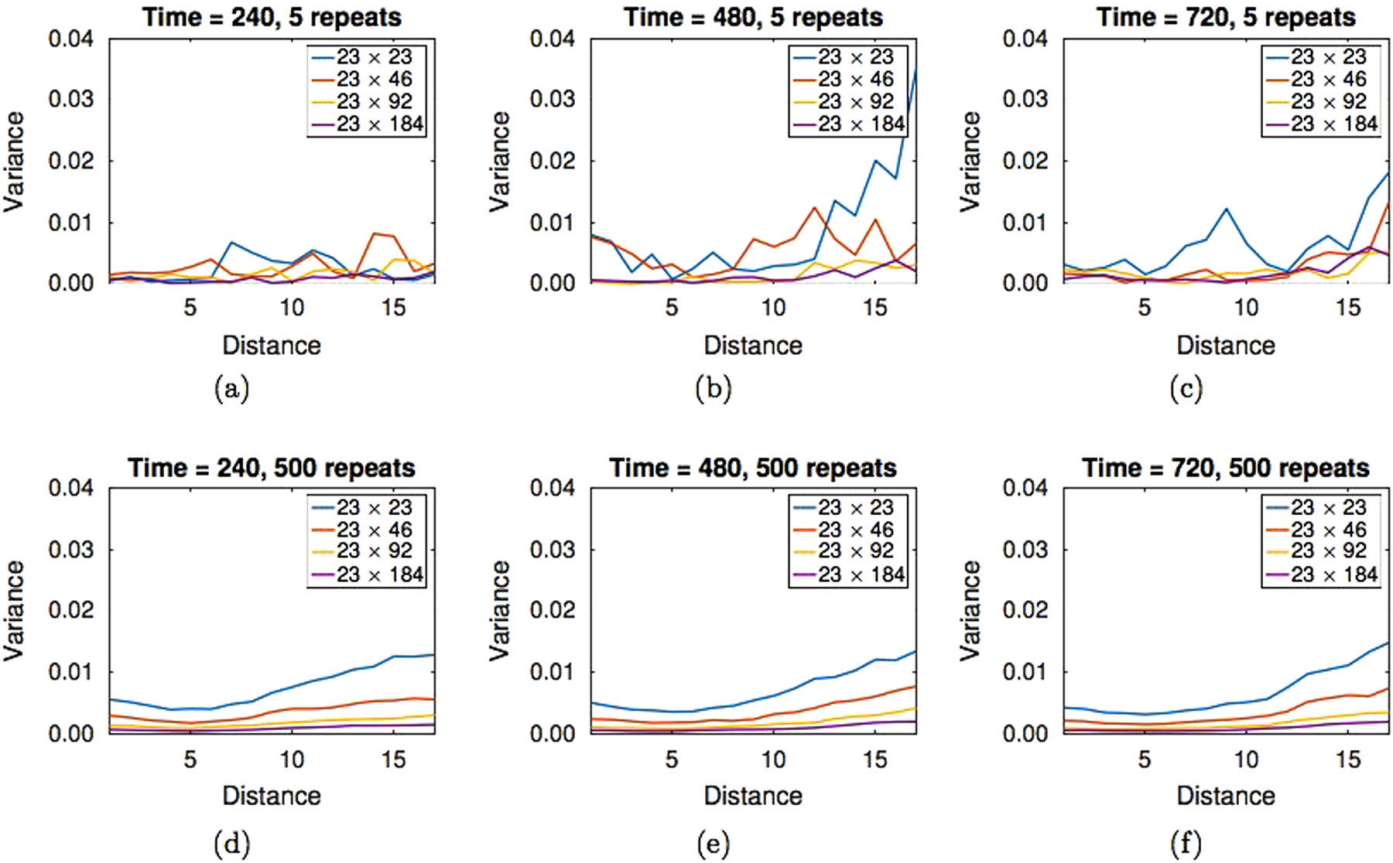
The variance in the PCF synthetic data for model B with *P*_*m*_ = *0.5*, *α* = 0.25 and different ABM domain sizes. Panels **a–c** display synthetic data generated from five replicates of the ABM, panels **d–f** display synthetic data generated from 500 replicates of the ABM. The domain size is indicated in the legend

A possible reason why the synthetic data parameters cannot be identified is that the synthetic data does not accurately represent the parameter values used to generate it, making parameter identification infeasible. To examine this possibility, we calculated the variance in the PCF synthetic data. In Fig. 4a–c the blue line indicates the variance in the PCF synthetic data for the current simulation design generated from five replicates of the ABM on a domain of dimension *L*_*x*_ = 23 by *L*_*y*_ = 23.

If the variance in the summary statistics of the synthetic data precludes accurate identification of model parameters using ABC, a sensible strategy may be to examine methods to reduce the variance in the summary statistics of the synthetic data. Reducing the variance of the summary statistics may mean the synthetic data is a more accurate reflection of the parameters values used to generate it. This may also explain why parameter identification for the unrealistic experiment was successful, as the variance in the summary statistics of the synthetic data was much smaller than for the practically realisable experiment (data not shown).

**Fig. 5:**
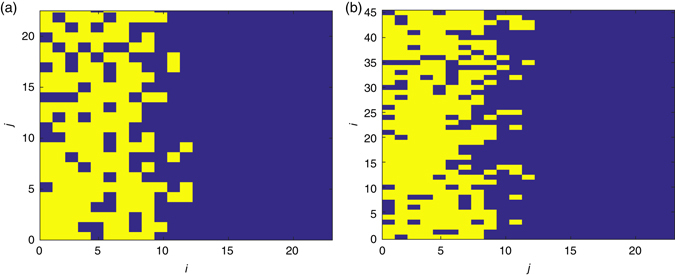
Increasing the size of the simulation domain while keeping the initial column densities the same. The domain in **b** is twice the size of that in **a**, however, the average initial density of each column is the same in both **a** and **b**

We conjectured that the variance in the summary statistics of the synthetic data could be reduced in two ways:

1. increasing the number of ABM replicates used to generate the synthetic data;

2. increasing the size of the ABM domain while keeping the column density of the initial conditions invariant. An example of this proposed initial condition is given in Fig. 5b, in which the domain is twice the size of that in Fig. 5a. Importantly, increasing the size of the ABM domain increases the number of agents in the simulation, and can be thought of as equivalent to increasing the field of view of the microscope.

In Fig. 4, the variance in the PCF synthetic data for model B with *P*_*m*_ = 0.5 and *α* = 0.25 for different domain sizes and varying numbers of replicates can be seen. It is evident that the variance in the PCF calculated from 500 replicates of our ABM on a *L*_*x*_ = 23 by *L*_*y*_ = 23 sized domain (blue line in Fig. 4d–f) is greater than the variance in the PCF calculated from five replicates of our ABM on a *L*_*x*_ = 23 by *L*_*y*_ = 184 sized domain (purple line in Fig. 4a–c). This can be understood by considering Eq. 9: the number of occupied lattice pairs for each horizontal pair distance used to generate the PCF does not increase linearly with the number of agents. Specifically, the number of occupied lattice pairs for each horizontal pair distance that generates the PCF is proportional to (This is not quite correct as a distance of ‘0’ between agents, that is they share the same column, is not accounted for in Eq. 9. To make Eq. 2 exact is not trivial as the expected number of agents each agent shares a column with depends on both the column position and simulation time.)

2 $$\frac{{N(N - 1)}}{2}.$$

Therefore, the identification of parameters in experimental data using the PCF as a summary statistic may be best facilitated by increasing the size of the domain upon which the experiment is performed, rather than increasing the number of replicates of an experiment with a smaller domain. Further variance plots for models A and B for the PCF summary statistic can be found in the supplementary material (Section S6).

It is important to note that it is also the case for the *agent density profile* synthetic data, that increasing the size of the domain is more effective at reducing variance in the synthetic data than increasing the number of replicates. If generated from 500 replicates of our ABM on a *L*_*x*_ = 23 by *L*_*y*_ = 23 sized domain, the agent density profile synthetic data will have greater variance than the agent density profile synthetic data generated from five replicates of our ABM on a *L*_*x*_ = 23 by *L*_*y*_ = 184 sized domain (data not shown). In this case, the reduction in variance is an artefact of the lattice-based model. This is because the density of each column in the ABM can take on a greater range of values between 0 and 1 as the column length is increased, leading to a reduction in variance in the agent density profile synthetic data (especially in the initial conditions of the simulations used to generate the synthetic data). However, as we do not use the agent density profile summary statistic to identify parameters in the current simulation design we do not pursue this matter further.

### Improving the experimental design

We now confirm that more accurate identification of synthetic data parameters can be carried out by expanding the domain upon which the experiment is performed, as opposed to increasing the number of experimental replicates.

**Fig. 6:**
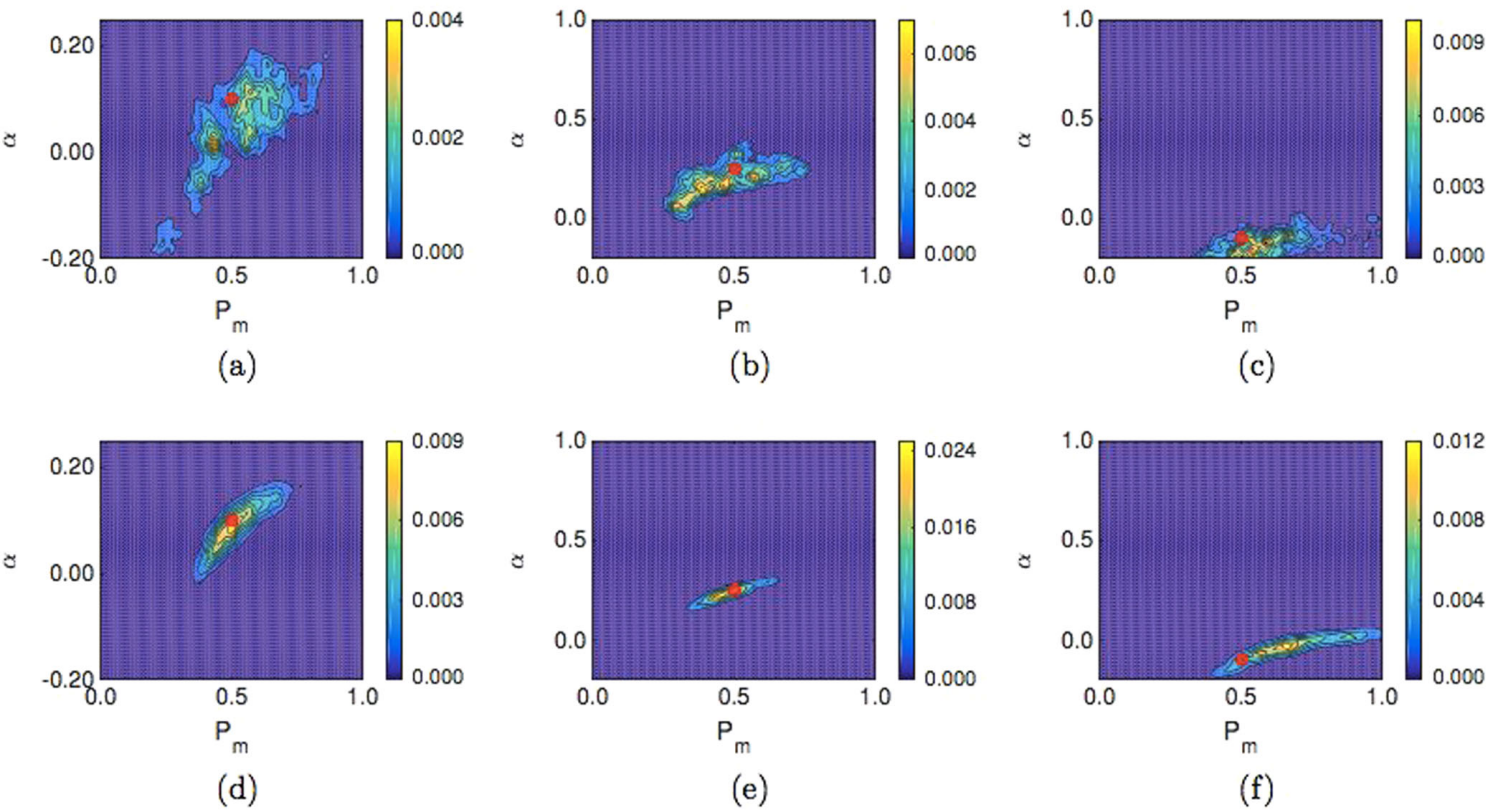
**a–c** Posterior distributions for simulations of the realistic experiment using the PCF as a summary statistic for an ABM simulated on a domain of dimension *L*_*x*_ = 23 by *L*_*y*_ = 23 with synthetic data generated from 500 replicates. **a** Model A: *P*_*m*_ = 0.5, *α* = 0.1, **b** model B: *P*_*m*_ = 0.5, *α* = 0.25, **c** model B: *P*_*m*_ = 0.5, *α* = −0.1. **d–f** Posterior distribution plots for simulations of the experiment using the PCF as a summary statistic for an ABM simulated on a domain of size *L*_*x*_ = 23 by *L*_*y*_ = 184 with synthetic data generated from five replicates. **a** Model A: *P*_*m*_ = 0.5, *α* = 0.1, **b** model B: *P*_*m*_ = 0.5, *α* = 0.25, **c** model B: *P*_*m*_ = 0.5, *α* = −0.1. Further figure information can be found in Fig. 1

**Fig. 7:**
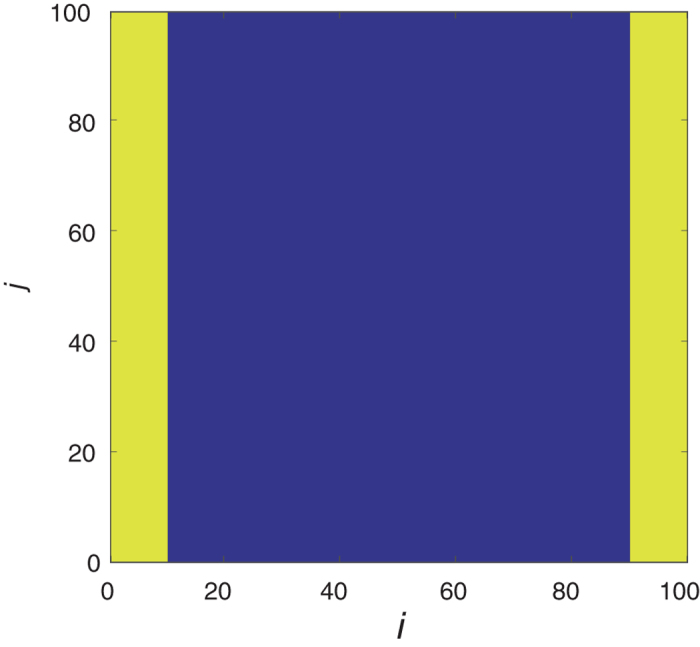
The initial condition in the ABM for the unrealistic experiment. *Yellow* indicates a site occupied by an agent and *blue* indicates an empty lattice site

In Fig. 6a–c, we plot the posterior distribution for synthetic data generated from 500 replicates of our ABM on a *L*_*x*_ = 23 by *L*_*y*_ = 23 sized domain, while in Fig. 6d–f we plot the posterior distribution generated from synthetic data generated from five replicates of our ABM on a *L*_*x*_ = 23 by *L*_*y*_ = 184 sized domain (A Markov chain Monte Carlo trace corresponding to Fig. 7e can be found in the supplementary material (Section S5)). As predicted, it is apparent that increasing the domain size is more effective for parameter identification than increasing the number of replicates used to generate the synthetic data. This is evident in the location (and narrow spread) of the posterior distribution relative to the red dot, whereby the peak of the posterior distribution is closer to the red dot in the case of Fig. 6d–f compared to Fig. 6a–c. Despite this, the identification of the parameters for repulsive interactions remains somewhat elusive (Fig. 6f). A possible reason for this is that the repulsive interaction we present here is a weak one, due to the constraint of Eqs. 4 and 6, and larger values of |*α*| are easier to identify as they have a more profound effect on the behaviour of the agent population.

Computing *D*_*KL*_(*p|π*) for all six plots in Fig. 6 gives: (a) 2.55; (b) 2.69; (c) 1.53; and (d) 3.69; (e) 2.97; (f) 3.54. In tandem with the proximity of the peak of the posterior distribution densities to the red dots in Fig. 6d–f compared to Fig. 6a–c, this increase in the KLD suggests that generating synthetic data on a larger domain is more effective for improving parameter identification than increasing the number of replicates used to generate the synthetic data.

## Discussion

In this work, we have presented methods to identify motility and adhesion parameters in an ABM of a wound-healing assay. Our findings suggest that for a commonly performed experiment increasing the size of the experimental domain can be more effective in improving the accuracy of parameter identification, when compared to increasing the number of replicates of the experiment. This is because increasing the size of the domain, which is equivalent to increasing the number of cells in the experiment, more effectively reduces the variance in the summary statistics of the synthetic data from which the parameters are identified. The reason for this reduction in variance is explained by Eq. 9, where the number of agent pair counts that generate the PCF increases nonlinearly with the number of agents on the domain. In addition, increasing the size of the experimental domain may make the collection of experimental data less time-consuming, as potentially fewer replicates of the experiment will have to be conducted. For instance, five replicates of the experiment on a larger domain provides more information about parameters than 500 replicates of the experiment on a smaller domain (in the examples we have presented in this work). Therefore, a comprehensive study of all summary statistics commonly used for analysing cell migration, to understand how their variance scales with the size of the experimental domain, is an interesting avenue for further research.

We also studied using the average horizontal displacement of agents and the agent density profile as summary statistics. These were found to be less effective than the PCF in parameter identification. This was especially the case for the averaged agent displacement, whereby a range of adhesion and motility parameters could result in the same average agent displacement. This result suggests that agent displacement may not be a suitable summary statistic for identifying cell motility and adhesion parameters, due to parameter identifiability issues.

The most obvious extension to the work presented here is to experimentally validate the findings. That is, expand the wound-healing experimental domain and demonstrate: (i) the cell migratory process can be effectively described by the model we have presented here; and (ii) the experimental parameters are identifiable given a larger experimental domain. If validated, evidence may be provided that demonstrates which adhesion model, A or B, is more applicable to the cell type under consideration. Subsequently, we could add further agent behaviours to the ABM, such as the role of the cell cycle. This may allow us to better capture the behaviour of the cell populations we have studied here, and so produce more realistic models of cell migration.

To conclude, the findings presented in this work will be of particular interest to those concerned with performing experiments that enable the effective parameterisation of cell migratory processes. In particular, cell migratory processes in which cell–cell adhesion or repulsion are known to play an important role. More generally, we have also suggested time and cost-saving alterations to a commonly performed experiment for identifying cell motility parameters.

## Methods

In this section, we first introduce the ABM. We then define our summary statistics and explain ABC and its implementation.

### Agent-based model

An ABM is a computational model for simulating the behaviour of autonomous agents. The agents in the ABM represent cells, and each agent is able to move and interact with other agents. The ABM is simulated on a two-dimensional square lattice with lattice spacing^31^ and size *L*_*x*_ by *L*_*y*_, where *L*_*x*_ is the number of lattice sites in each row, and *L*_*y*_ is the number of sites in each column. Each agent is initially assigned to a lattice site, from which it can move into adjacent sites. If an agent attempts to move into a site that is already occupied by another agent, the movement event is aborted. Processes such as this whereby one agent is allowed per site are often referred to as exclusion processes.^31^ In the ABM time evolves continuously, and as our ABM can be modelled as a continuous-time Markov process we use the Gillespie algorithm^32^ to generate sample paths. Attempted agent movement events occur with rate *P*_*m*_ per unit time. *P*_*m*_ *δt*, therefore, is the probability of an agent attempting to move in the next infinitesimally small time interval *δt*. In our ABM, a lattice site is denoted by *v* *=* (*i*, *j*), where *i* indicates the column number and *j* the row number. Each lattice site has four adjacent lattice sites (except for those sites situated on nonperiodic boundaries), and so the number of nearest neighbour lattice sites that are occupied by an agent, denoted by *n*, is 0 ≤ *n* ≤ 4. We denote the set of unoccupied nearest neighbour lattice sites by *U*.

The ABM domain size for simulations representing unrealistic experiments is *L*_*x*_ *=* 100 by *L*_*y*_ *=* 100, and the lattice sites indexed by 1 ≤ *j* ≤ *L*_*y*_ and 1 ≤ *i* ≤ 10, and1 ≤ *j* ≤ *L*_*y*_ and 91 ≤ *i* ≤ *L*_x_ are initially occupied by agents. In Fig. 7, the initial conditions in the ABM for the unrealistic experiment can be seen. The initial condition in Fig. 7 represents a ‘wound’, in that agents are positioned either side of a space, the ‘wound’, that they can migrate into. The agent migration into this space simulates one aspect of the wound-healing process. We refer to this simulation as unrealistic because the uniformity of the initial conditions would not be possible in a realistic experimental setting. The initial condition is also improved from our experimentally realisable simulation as it is ‘double-sided’, as opposed to the ‘single-sided’ experimental data that we will later simulate for our ABM of a realistic experiment. It has been shown that double-sided initial conditions can provide more information than single-sided initial conditions for some model parameters.^11^ For instance, double-sided initial conditions can improve parameter identifiability if increasing the number of agents in a simulation improves parameter identifiability.

For the ABM of an unrealistic experiment, all simulations have periodic boundary conditions at the top and bottom of the domain (i.e. for lattice sites indexed by *j* *=* 1 or *j* *=* *L*_*y*_), and no-flux boundary conditions at the left-hand and right-hand boundaries of the domain (i.e. for lattice sites indexed by *i* *=* 1 or *i* *=* *L*_*x*_).

It is important to stress that throughout this work we assume that cellular processes such as migration have constant parameter values associated with them. Inference procedures do exist in which the parameter values associated with cell processes are not assumed to be constant, but are instead treated as a random variable sampled from a distribution. These methods are often important for sensitivity analysis, or if the data is sampled from a heterogeneous population.^33–35^ However, we do not implement these methods in this work as it would serve to prematurely complicate our research question. It is also important to acknowledge that in migrating cell populations there are often many more factors at play than simply cell motility and adhesion. For instance, the cell cycle and a cell’s response to environmental cues may be important factors in a cell’s behaviour. Again, however, we have purposely simplified our model to first ascertain if we can accurately estimate parameters associated with cell motility and adhesion.

### Cell–cell adhesion models

In the ABM cell–cell interactions are simulated by altering the probability of an agent attempting to move, depending on the number of nearest occupied neighbours, *n*, an agent has. We employ two models to simulate cell–cell interactions in the ABM, one of which has been published before.^13, 36^ We define *T*(*v*′|*v*) as the transition probability that an agent situated at site *v*, having been selected to move, attempts to move to site *v*′, where *v*′ indicates one of the nearest neighbour sites of *v*. Therefore, *T*(*v*′|*v*) is only non-zero if *v* and *v*′ are nearest neighbours. The transition probability in the first model, which we refer to as model A, is defined as

3 $${T_{\rm{A}}}\left( {v\prime \left| v \right.} \right) = \frac{{1 - n\alpha }}{4},$$

where *α* is the adhesion parameter. The subscript A on the transition probability in Eq. 3 indicates that this is the transition probability for model A. If *α* *>* 0 Eq. 3 models cell–cell adhesion, and if *α* *<* 0 Eq. 3 models cell–cell repulsion. The transition probabilities stated in Eq. 3 must satisfy

4 $$0 \le \mathop {\sum}\limits_{v\prime \in U}^U {{T_{\rm{A}}}\left( {v\prime \left| v \right.} \right)} \le 1.$$

Inequality (4) ensures the probability of an agent, if selected to move, attempting to move to any of its unoccupied nearest neighbour sites never exceeds unity, and so constrains the value *α* can take. The transition probability in the second model, which we refer to as model B,^13, 36^ is defined as

5 $${T_{\rm{B}}}\left( {v\prime \left| v \right.} \right) = \frac{{{{\left( {1 - \alpha } \right)}^n}}}{4},$$

and must satisfy

6 $$0 \le \mathop {\sum}\limits_{v\prime \in U}^U {{T_{\rm{B}}}\left( {v\prime \left| v \right.} \right)} \le 1.$$

As in model A if *α* *>* 0 Eq. 5 models cell–cell adhesion, and if *α* *<* 0 Eq. 5 models cell–cell repulsion.

Models A and B simulate different types of cell–cell adhesion. In model A, the transition probability is a linear function of *n*. Meanwhile, in model B the transition probability is a nonlinear function of *α*. Not only may these different types of cell–cell adhesion be relevant for different cell types, but implementing two models of cell–cell adhesion allows us to test the robustness of the methods we present in this work for identifying cell–cell adhesion parameters.

### Summary statistics

Summary statistics are lower-dimensional summaries of data that provide a tractable means to compare different sets of data. Summary statistics are important because experimental data is often of high dimensionality, and if we want to use experimental data to efficiently guide computational algorithms we require ways to accurately summarise it. We now define the summary statistics we apply to the ABM output and experimental data. Following this, we describe how we utilise these summary statistics to implement ABC.

We initially use three summary statistics to evaluate the ABM output, all of which have been considered previously.^9, 36, 37^ Our aim is to ascertain which summary statistic (or combination of summary statistics) is most effective for the identification of agent motility and adhesion parameters in the ABM.

### Average horizontal displacement of agents

The average horizontal displacement of all agents, *ī*, in a given time interval, [*t*_i_, *t*_*f*_], in the ABM is calculated as

7 $$\bar i = \frac{1}{N}\mathop {\sum }\limits_{k = 1}^N \left| {i_{{t_i}}^k - i_{{t_f}}^k} \right|,$$

Where *ī* is the average horizontal displacement of agents, *N* is the total number of agents in the simulation, $\mathop {i}\nolimits_{{t_i}}^k$ is the column position of agent *k* at time *t*_*i*_, and $\mathop {i}\nolimits_{{t_f}}^k$ is the column position of agent *k* at time *t*_*f*_. We only look at the horizontal displacement of agents as this is the direction in which the majority of agent displacement occurs, due to the initial conditions of the ABM (Fig. 7). It has previously been shown that different cell–cell interactions have different effects on the average displacement of agents in an ABM.^36^ As may be expected, repulsive (adhesive) interactions between agents tend to increase (decrease) the average displacement of agents, and so the average displacement of agents may be a useful summary statistic for distinguishing between repulsive and adhesive cell–cell interactions in the ABM.

### Agent density profile

The agent density profile at time *t* in the ABM is calculated as

8 $${C_t}\left( i \right) = \frac{1}{{{L_y}}}\mathop {\sum }\limits_{j = 1}^{{L_y}} {\bf{1}}\left\{ v \right\}.$$

Here *C*_*t*_(*i*) is the agent density profile and **1** is the indicator function for the occupancy of a lattice site *v* (i.e., 1 if an agent occupies lattice site *v*, and 0 if it is not occupied by an agent). We have shown previously that different cell–cell interactions have different effects on the agent density profile.^36^ For instance, repulsive interactions between agents can create a concave agent density profile, whereas adhesive interactions between agents can create a convex agent density profile. Therefore, the agent density profile may be an effective summary statistic for distinguishing between repulsive and adhesive cell–cell interactions in the ABM.

### Pairwise-correlation function

The final summary statistic, we consider is the pairwise-correlation function (PCF). The PCF provides a measure of the spatial clustering between agents in an ABM, and has been used frequently in the analysis of cell migratory processes.^4, 9, 38, 39^ The PCF has also been successfully used as a summary statistic for the parameterisation of ABMs of cell migration.^10^ We use $\mathop {i}\nolimits_t^k$ to denote the column position of agent *k* at time *t*, $\mathop {i}\nolimits_{{t_{}}}^l$ to denote the column position of agent *l* at time *t*, and define *c*_*t*_(*m*) to be the number of occupied pairs of lattice sites for each *nonperiodic* (By nonperiodic it is meant the distance measured between two agents cannot cross the ABM boundary) horizontal pair distance *m* *=* 1,…,*L*_*x*_−1 at time *t*. This means *c*_*t*_(*m*) is given by

9 $${c_t}\left( m \right) = \mathop {\sum }\limits_{k = 1}^N \mathop {\sum }\limits_{l = k + 1}^N {\bf{1}}\left\{ {\left| {\mathop {i}\nolimits_t^k - \mathop {i}\nolimits_t^l } \right| = m} \right\},\,\forall \,m = 1, \ldots ,{L_x} - 1,$$

Where **1** is the indicator function equal to unity if $\left| {\mathop {i}\nolimits_t^k - \left. {\mathop {i}\nolimits_t^l } \right|} \right.$ *=* *m*, and is equal to zero otherwise. In Eq. 9 only the pair agent distances in the horizontal direction are counted. Given the translational invariance of the initial conditions in the vertical direction of the ABM, the majority of important spatial information will be in the horizontal direction (This approach is in agreement with previous studies^39^, which showed the most relevant information from the PCF summary statistic is perpendicular to the wound axis in a wound-healing assay). Binder and Simpson^39^ demonstrated that is necessary to normalise Eq. 9 to account for volume exclusion. The normalisation term is

10 $${\hat c_t}\left( m \right) = L_y^2\left( {{L_x} - m} \right)\rho \hat \rho ,\,\forall \,m = 1, \ldots ,{L_x} - 1,$$

where = *N*/(*L*_*x*_ *L*_*y*_) , and $\hat \rho$ = (*N*−1)/(*L*_*x*_ *L*_*y*_ *−*1). Eq. 10 describes the expected number of pairs of occupied lattice sites, for each nonperiodic horizontal pair distance, *m*, in a population distributed uniformly at random on the domain. Combining Eqs. 9 and 10, the PCF is

11 $${q_t}(m) = \frac{{{c_t}(m)}}{{{{\hat c}_t}(m)}},$$

Where *q*_t_(*m*), the PCF, is a measure of how far c_*t*_(*m*) departs from describing the expected number of occupied lattice pairs for each horizontal distance of an agent population spatially distributed uniformly at random on the ABM domain.

It is important to briefly discuss why we chose these summary statistics and not others that have also been used to analyse cell migration.^10, 22, 24^ Other summary statistics were initially implemented in this study, such as the concavity of agent trajectories, the *total* distance travelled by agents and the leading edge of the agent population. However, these summary statistics were found not to be informative for the identification of agent motility and adhesion parameters in our ABM, and so were excluded from this work. The three summary statistics we implement are encapsulated in Table 1 for the reader’s convenience, in addition to the properties each summary statistic summarises in the agent population.

**Table 1:** The summary statistics we implement and the properties of the agent population they summarise

Summary statistic	Description
Average horizontal displacement of agents	Summarises the displacement of agents into the ‘wound’. This displacement is affected by the adhesion of agents and their motility rate. Mathematically the average horizontal displacement of agents is defined as $\bar i = \frac{1}{N}\mathop {\sum }\nolimits_{k = 1}^N \left| {i_{{t_i}}^k - i_{{t_f}}^k} \right| \cdot$
Agent density profile	Summarises the macroscopic shape of the population as it moves into the ‘wound’. We have previously shown this shape is partly determined by agent interactions and motility.^36^ Mathematically, the agent density profile is defined as ${C_t}\left( i \right) = \frac{1}{{{L_y}}}\mathop {\sum }\nolimits_{j = 1}^{{L_y}} {\bf{1}}\left\{ v \right\}.$
Pair-wise correlation function	Summarises the spatial correlations/structure established by agent movement and interactions. Mathematically the pair-wise correlation function is defined as ${q_t}\left( m \right) = \frac{{{c_t}\left( m \right)}}{{{{\hat c}_t}\left( m \right)}} \cdot$

### Approximate Bayesian computation

Here, we introduce our ABC algorithm.^19^ We define *M* as a stochastic model that takes parameters *Θ* and produces data *D*. This relationship can be written as *D*~*M*(*Θ*). For the ABM presented in this work *Θ* = (*P*_*m*_, *α*), where *Θ* is sampled from a prior distribution, *π*, and so this relationship can be written as *Θ~π*. The relationship between *π* and *Θ* is often written as ~π(*Θ*), which indicates that a new *Θ* sampled from the prior distribution may depend on the previous *Θ*. This relationship will be relevant later on in this work, however, initially each *Θ* sampled from *π* is independent of the previous *Θ*.

The identification of ABM parameters in this work centres around the following problem: given a stochastic model, *M*, and data, *D*, what is the probability density function that describes *Θ* being the model parameters that produced data *D*? More formally, we seek to obtain a posterior distribution, *p*(*Θ|D*), which is the conditional probability of *Θ* given *D* (and the model, *M*).

Typically, to compute the posterior distribution a likelihood function, *L*(*D*|*Θ*), is required. This is because the likelihood function and posterior distribution are related in the following manner by Bayes’ theorem:

12 $$p\left( {{\it{\Theta }}{\rm{|}}D} \right) \propto L\left( {D{\rm{|}}{\it{\Theta }}} \right)\pi \left( {\it{\Theta }} \right).$$

That is, the posterior distribution is proportional to the product of the likelihood function and the prior distribution.

ABC is a well-known method for estimating posterior distributions of model parameters in scenarios where the likelihood function is *intractable* i.e., it is impossible or computationally prohibitive to obtain.^19^

In many cases for ABC, due to the high dimensionality of the data, *D*, it is necessary to utilise a summary statistic, *S* = *S*(*D*). The summary statistics we employ in this work are of varying dimension. For instance, the agent density profile at time *t* has *L*_*x*_ data points, whereas the average agent displacement at time *t* has one data point. Therefore, we write *S*(*D*) as S(*D*)_*r,t*_, where *S*(*D*)_*r,t*_ is the *r* ^*th*^ data point in the summary statistic at the *t* ^*th*^ sampling time.

The ABC method proceeds in the following manner: we wish to estimate the posterior distribution of *Θ* given *D*. We now simulate model *M* with parameters *Θ*, sampled from *π*, and produce data $\tilde D$. We calculate the difference between a summary statistic applied to *D* and $\tilde D$ with

13 $$d = {\mathop {\sum}\limits_{t = 1}^T {\mathop {\sum}\limits_{r = 1}^R {\left| {S{{(D)}_{r,t}}} \right. - S\left( {\tilde D} \right)_{r,t}}}} \left|, \right.$$

Where *R* is the number of data points in *S*(*D*) and *T* is the number of sampling times. We repeat the above process many times, that is, sample *Θ* from *π*, produce $\tilde D$, calculate *d* with Eq. 13, and only accept *Θ* for which *d* is below a user defined certain threshold (alternatively, a predefined number of *Θ* that minimise *d* can be accepted). This enables us to generate a distribution for *Θ* that is an approximation of the posterior distribution, *p*(*Θ|D*), given *M*.^40^ More specific details of the ABC algorithms we implement are introduced when necessary in the text.

## Electronic supplementary material

Supplementary Information
