# Supplementary material for: Using approximate Bayesian computation to quantify cell–cell adhesion parameters in a cell migratory process
Source: NPJ Syst Biol Appl. 2017 Mar 10;3:9. doi: 10.1038/s41540-017-0010-7 (PMC5445583; doi:10.1038/s41540-017-0010-7)
Supplement: Supplementary file 1 — Supplementary Information [file 41540_2017_10_MOESM1_ESM.pdf]

Supplementary information: Using approximate Bayesian computation to quantify cell-cell adhesion parameters in a cell migratory process

Robert J. H. Ross *[1], R. E. Baker †[1], Andrew Parker ‡[1], M. J. Ford §[2], R. L. Mort ¶[3], and C. A. Yates ‖[4]

[1]Wolfson Centre for Mathematical Biology, Mathematical Institute, University of Oxford, Radcliffe Observatory Quarter, Woodstock Road, Oxford, OX2 6GG

[2]MRC Human Genetics Unit, MRC IGMM, Western General Hospital, University of Edinburgh, Edinburgh, EH4 2XU

[3]Division of Biomedical and Life Sciences, Faculty of Health and Medicine, Furness Building, Lancaster University, Bailrigg, Lancaster, LA1 4YG

[4]Centre for Mathematical Biology, Department of Mathematical Sciences, University of Bath, Claverton Down, Bath, BA2 7AY

January 17, 2017

*ross@maths.ox.ac.uk
†baker@maths.ox.ac.uk
‡parker@maths.ox.ac.uk
§matthew.ford@ed.ac.uk
¶r.mort@lancaster.ac.uk
‖c.yates@bath.ac.uk

S1: Combining summary statistics

In Fig. S1 we plot the posterior distribution generated from combining all three summary statistics[1]. As described in the main text, there is little difference between Fig. S1 in the supplementary material and Fig. 7 (e) of the main text. We quantify the difference between the posterior distributions in Fig. S1 and Fig. 7 (e) as follows:

$$\text{Difference} = \frac{1}{N}\sum_{n}^{N} |p^A(\Theta_n|D) - p^B(\Theta_n|D)|, \tag{S1}$$

where the index n accounts for all possible discretised parameter pairs, $p^A(\Theta_n|D)$ is the posterior distribution in Fig. S1, and $p^B(\Theta_n|D)$ is the posterior distribution in Fig. 7 (e). The difference between the posterior distributions in Fig. S1 and Fig. 7 (e) is 0.00006, which shows that the performance of all three summary statistics is little different from the performance of the PCF summary statistic individually. By means of comparison the difference between the posterior distributions in Fig. 7 (b) and Fig. 7 (e) is 0.00031.

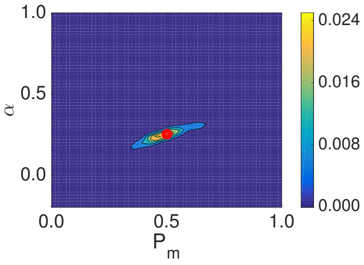

(a)

Figure S1: Posterior distribution plot for simulations of the experiment using all three summary statistics for an ABM simulated on a domain of dimension $L_x = 23$ by $L_y = 184$ with synthetic data generated from five replicates. Model B: $P_m = 0.5$, $\alpha = 0.25$.

[1]To combine all three summary statistics we implement Eq. (11) (equivalently Eq. (S3)). If the condition stipulated in Section S4 fails for any individual summary statistic the parameter values are rejected.

S2: Experimental methods

The details of the experiment we aim to identify cell motility and adhesion parameters from is as follows: Fucci2a 3T3 flp-In cells were maintained in dulbeccos modified eagle medium (DMEM) containing 10% fetal calf serum, 1% Penicillin/Streptomycin and 100µg/ml Hygromycin B [1]. A silicon well (Ibidi) was attached to the surface of a 24-well glass-bottomed plate (Greiner bio-one) by surface tension and allowed to attach overnight. Cells were plated within the insert and allowed to attach phenol-red free DMEM (Biochrom) containing 10% fetal calf serum, and 1% Penicillin/Streptomycin. Cells migrating from the leading edge of the cell mass were then imaged with a 20x objective using a Nikon A1R inverted confocal microscope in a heated chamber supplied with 5% CO2 in air. All image analysis tasks (required to generate the initial conditions for the ABM of a practically realisable experiment) were performed using custom written macros for the Fiji [2] distribution of ImageJ an open source image analysis package based on NIH Image [3]. The cell nucleus of each cell was identified by merging of the green and red channels containing the Fucci signal followed by segmentation. The centre of mass of each object in the segmented image was then determined automatically.

In total we have data from five replicates of the experiment. Each data set contains cell track data for every cell for sixty-four hours imaged at twenty minute intervals. Therefore, we have the information required to apply our summary statistics to the experimental data. More specifically, we have the position of all cells at each time interval so that the expected horizontal displacement of cells, cell density profile, and PCF may be computed.

S3: Practically realisable experiment ABM design

Initial conditions

To map the position of cells in the experimental images where cell position is a continuous variable, (x, y), to a discrete lattice site, (i, j), we use the following formulae

$$i = \left\lceil \frac{x}{\Delta} \right\rceil, \quad j = \left\lceil \frac{y}{\Delta} \right\rceil, \tag{S2}$$

where $\lceil \cdot \rceil$ denotes the ceiling function and Δ is as defined in the main text. Given the experimental data and the lattice size no two cells were mapped to the same lattice site[2].

The application of Eq. (S2) to the initial frames of the five experiments allowed the average initial condition for the experimentally realistic ABM to be calculated. These initial conditions are expressed in terms of the average initial density of each column. These average initial column densities are:

Column	Initial density
1st	0.8261
2nd	0.7826
3rd	0.8261
4th	0.8261
5th	0.8261
6th	0.7391
7th	0.6957
8th	0.6087
9th	0.5217
10th	0.2609
11th	0.2174
12th	0.0870
$13^{th} - 23^{rd}$	0

[2] If two cells did map to the same lattice site, one of these cells would be placed in the nearest unoccupied lattice site to the original lattice site. If there was more than one nearest unoccupied lattice site, one of these sites would be chosen uniformly at random for the cell to be mapped to.

To generate the initial conditions at the start of each ABM realisation each site in a column receives an agent uniformly at random at a probability equal to the average initial column density of the column the site is in. Therefore, the initial condition in the ABM is generated such that an ensemble average of the initial conditions of many realisations would equal the averaged initial conditions from the experiment. This initial condition is then used in the experimentally realistic ABM simulations. An example of this initial condition can be seen in the main text.

Boundary conditions

Following the start of the simulation the density of the first column is checked after each agent movement event out of the first column in the ABM. If the first column's density is below 0.6, agents are added uniformly at random to empty sites in the first column until the density of the first column is greater than 0.6. This mechanism and density ensures that the agent density profile in the ABM matches the experimental density profile for the entire course of the experimental data throughout the simulation.

S4: Markov chain Monte Carlo ABC algorithm

We define a transition kernel w that proposes Θ' values as a bivariate uniform distribution. The transition kernel ensures $P_m \in [0, 1]$ and $\alpha \in [-0.2, 0.25]$ for the model A, and $P_m \in [0, 1]$ and $\alpha \in [-0.2, 1.0]$ for the model B. The parameter d^* is a constant selected so that approximately one percent of the proposed parameter sets are accepted, the value of which is obtained through trial and error.

To implement a Markov chain Monte Carlo method (Metropolis-Hastings algorithm) we proceed as follows [4]:

R1 If at Θ step to Θ' according to a transition kernel $w(\Theta \to \Theta')$.

R2 Simulate $\tilde{D}$ from the model using Θ' and calculate the summary statistic $S(\tilde{D})$ at each sampling point. That is, for each individual $t = [240, 480, 720]$ calculate d:

$$d = \sum_{r=1}^{R} |S(D)_{r,t} - S(\tilde{D})_{r,t}|, \tag{S3}$$

If $d > d^*$ (at any t) reject Θ' and return to R1.

R3 Calculate
$$h = \min\left(1, \frac{\pi(\Theta')w(\Theta' \to \Theta)}{\pi(\Theta)w(\Theta \to \Theta')}\right).$$

R4 Accept Θ' with probability h.

R5 Return to **1** until 10^6 steps have been attempted.

Initially, we sample Θ randomly from the prior distribution until a parameter set has been accepted (**R4**).

S5: Markov chains: trace plots

In Fig. S2 (d)-(i) the Markov chain traces for the posterior distributions for Fig. 4 in the main text are displayed. The mean and variance values for these chains are: (d) mean = 0.4722, variance = 0.0115; (e) 0.2202, 0.0050; (f) 0.6236, 0.0356; (g) -0.0377, 0.0102; (h) 0.0087, 0.0176; (i) -0.0734, 0.0074. The reason as to why the estimation of the values of P_m and α in the synthetic data is inaccurate in Fig. S2 is because the synthetic data (in conjunction with the PCF summary statistic) does not provide an accurate enough representation of the parameters with which the synthetic data was generated i.e. the parameters are not identifiable. Therefore, the Markov chain Monte Carlo ABC algorithm is not able to work effectively.

In the case of Fig. S3 (corresponds to Fig. 7 (e) in the main text) the same algorithm accurately estimates the parameter values used to generate the synthetic data. This is because the synthetic data in this case is an accurate representation of the parameters used to generate it. The mean and variance values for these chains are: (b) 0.5627, 0.0086; (c) 0.2718, 0.0017.

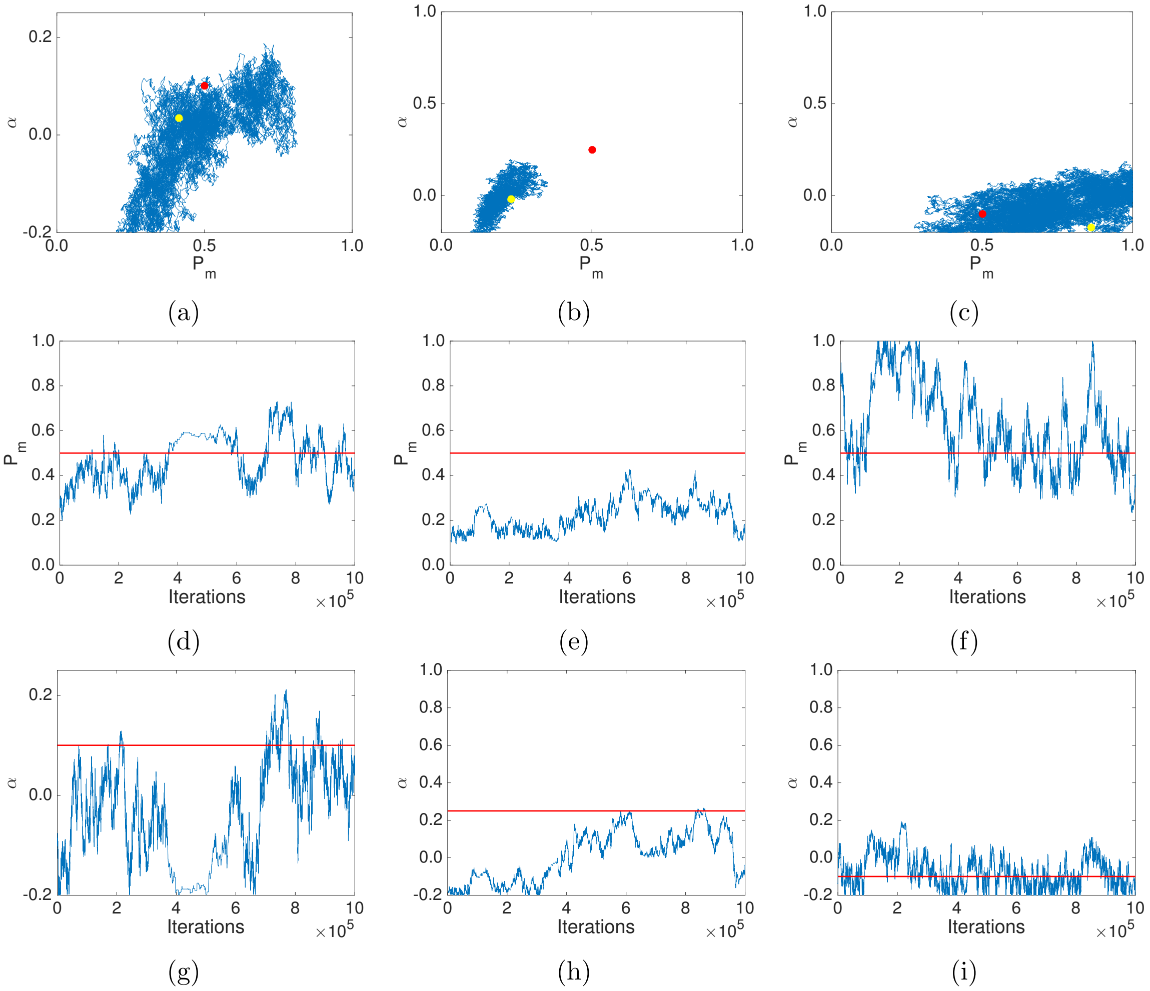

Figure S2: Markov chain Monte Carlo trace plots for Fig. 4 in the main text. In panels (a)-(c) the yellow dot indicates the initial value of the chain used to generate the posterior distributions, and the red dot indicates the parameter values used to generate the synthetic data. In panels (d)-(i) the red line indicates the value of the parameter used to generate the synthetic data. Panels (d)-(i) display individual parameter trace plots. Panels (a), (d) and (g) correspond to model A, $P_m = 0.5$, $\alpha = 0.1$. Panels (b), (e) and (h) correspond to model B, $P_m = 0.5$, $\alpha = 0.25$. Panels (c), (f) and (i) correspond to model B, $P_m = 0.5$, $\alpha = -0.1$.

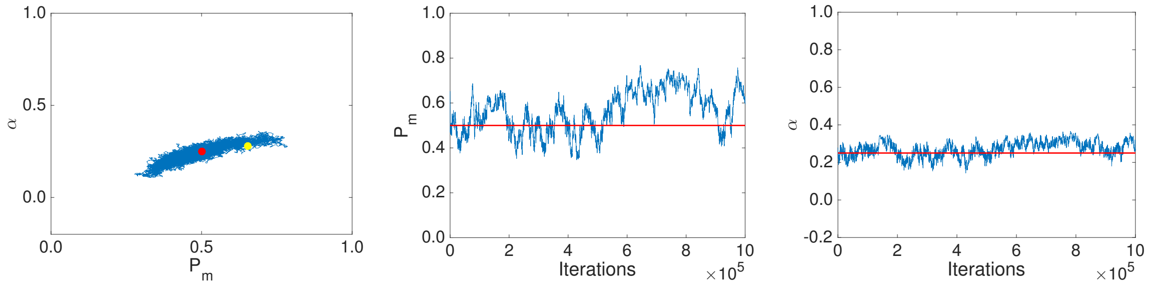

(a) (b) (c)

Figure S3: Markov chain Monte Carlo trace plots for Fig. 7 (e) in the main text. In panel (a) the yellow dot indicates the initial value of the chain used to generate the posterior distribution, and the red dot indicates the parameter values used to generate the synthetic data. In panels (b) and (c) the red line indicates the value of the parameter used to generate the synthetic data. Panels (b) and (c) display individual parameter trace plots. Panels (a), (b) and (c) correspond to model B, $P_m = 0.5$, $\alpha = 0.25$.

S6: Further variance plots for models A and B for the PCF summary statistic.

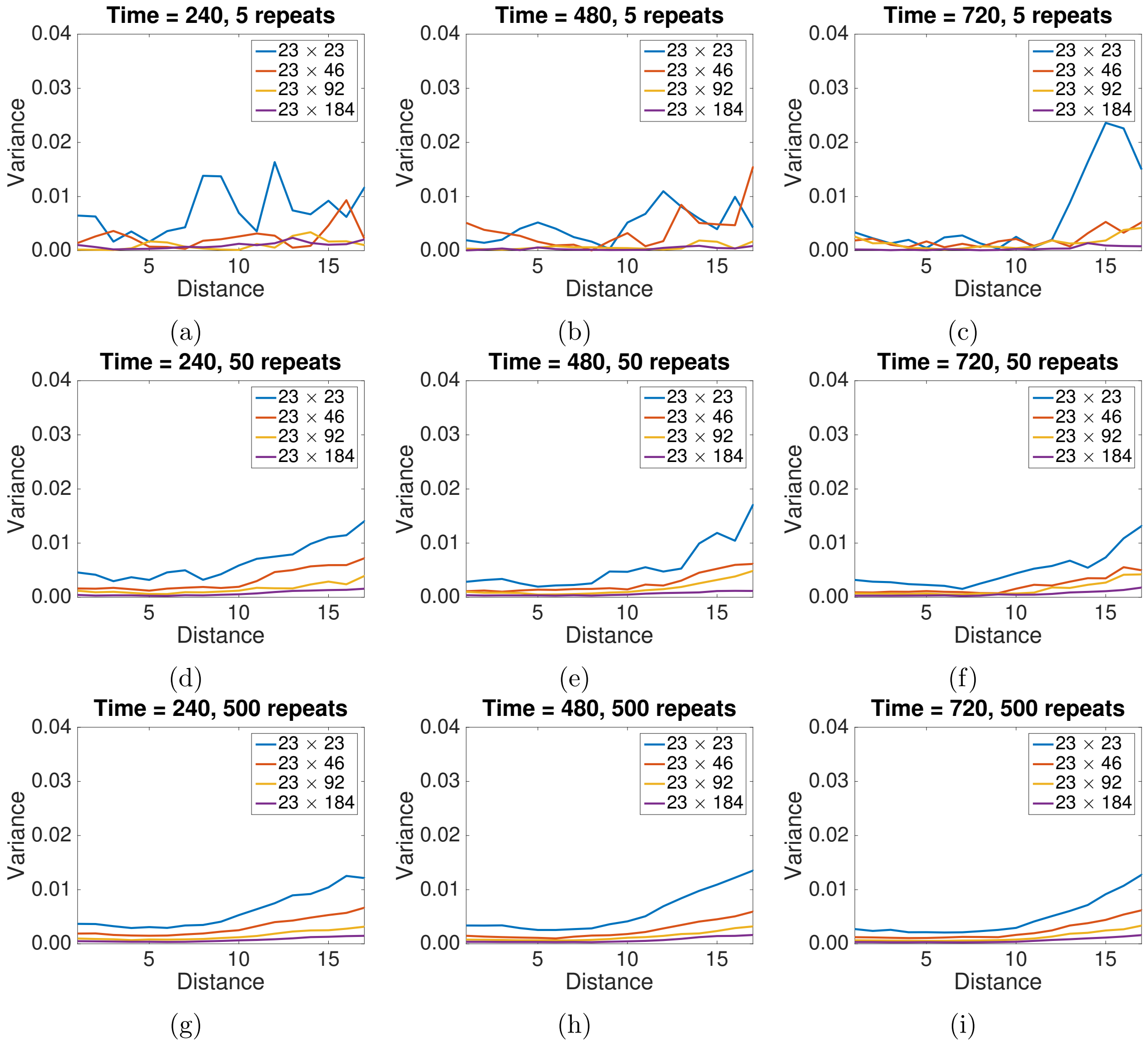

Figure S4: The variance in PCF synthetic data for model A with $P_m = 0.5$, $\alpha = 0.1$ for different ABM domain sizes. Panels (a)-(c) display synthetic data generated from five replicates of the ABM, panels (d)-(f) display synthetic data generated from 50 replicates of the ABM and panels (g)-(i) display synthetic data generated from 500 replicates of the ABM.

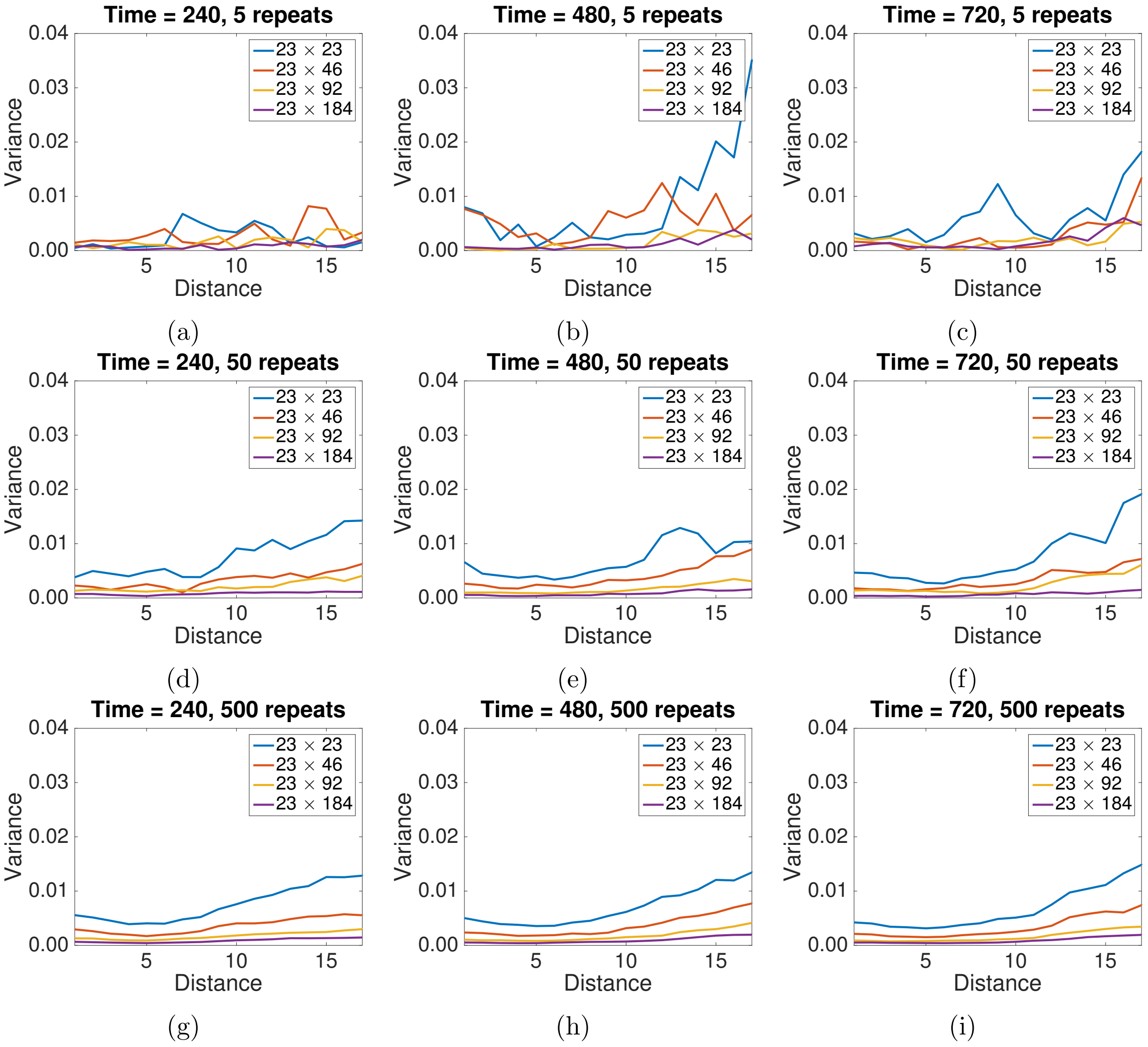

Figure S5: The variance in the synthetic data for model B with $P_m = 0.5$, $\alpha = 0.25$ for different ABM domain sizes. Panels (a)-(c) display synthetic data generated from five replicates of the ABM, panels (d)-(f) display synthetic data generated from 50 replicates of the ABM and panels (g)-(i) display synthetic data generated from 500 replicates of the ABM.

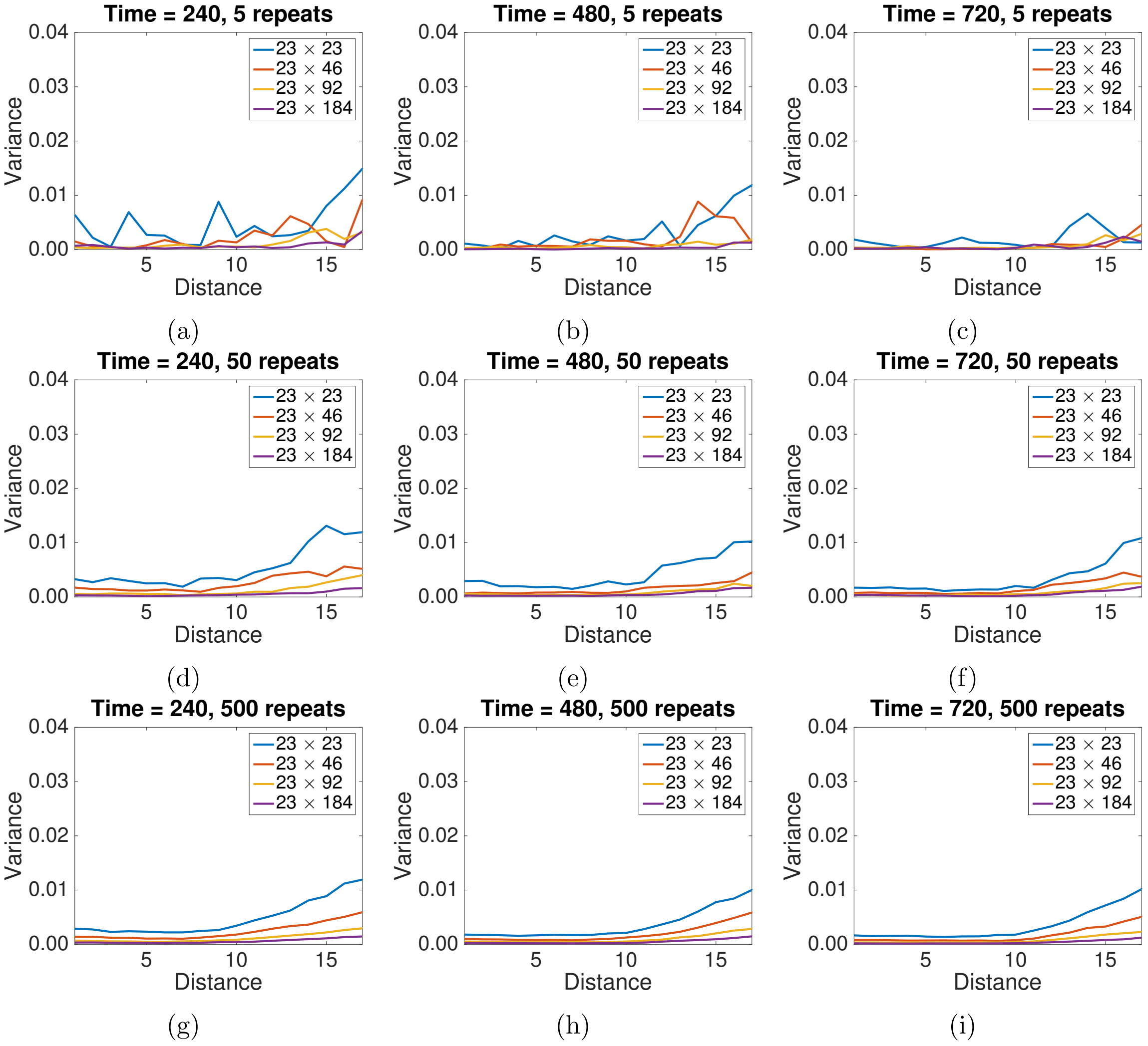

Figure S6: The variance in PCF synthetic data for model B with $P_m = 0.5$, $\alpha = -0.1$ for different ABM domain sizes. Panels (a)-(c) display synthetic data generated from five replicates of the ABM, panels (d)-(f) display synthetic data generated from 50 replicates of the ABM and panels (g)-(i) display synthetic data generated from 500 replicates of the ABM.

References

[1] R. L. Mort, M. J. Ford, A. Sakaue-Sawano, N. O. Lindstrom, A. Casadio, A. T. Douglas, M. A. Keighren, P. Hohenstein, A. Miyawaki, and I. J. Jackson. Fucci2a: a bicistronic cell cycle reporter that allows Cre mediated tissue specific expression in mice. *Cell Cycle*, 13 (17):2681–2696, 2014.

[2] J. Schindelin, I. Arganda-Carreras, E. Frise, V. Kaynig, M. Longair, T. Pietzsch, S. Preibisch, C. Rueden, S. Saalfeld, B. Schmid, J. Y. Tinevez, D. J. White, V. Hartenstein, K. Eliceiri, P. Tomancak, and A. Cardona. Fiji: an open-source platform for biological-image analysis. *Nature Methods*, 9(7):676–82, 2012.

[3] C. A. Schneider, W. S. Rasband, and K. W. Eliceiri. NIH image to ImageJ: 25 years of image analysis. *Nature Methods*, 9(7):671–5, 2012.

[4] P. Marjoram, J. Molitor, V. Plagnol, and S. Tavaré. Markov chain Monte Carlo without likelihoods. *Proceedings of the National Academy of Sciences*, 100(26):15324–15328, 2003.
